# Approach to Patients with Neurometabolic Diseases Who Show Characteristic Signs and Symptoms

**Published:** 2020

**Authors:** Parvaneh KARIMZADEH, Mohammad GHOFRANI, Shahram NASIRI

**Affiliations:** 1 Department of Pediatric Neurology, Pediatric Neurology Research Center, Research Institute for Children’s Health, Shahid Beheshti University of Medical Sciences, Tehran, Iran.; 2 Pediatric Neurology Department, Mofid Children’s Hospital, Faculty of Medicine, Shahid Beheshti University of Medical Sciences, Tehran, Iran; 3 Department of Paediatric Neurology, Abuzar children’s medical center, Ahvaz Jundishapur University of Medical Sciences, Ahvaz, IranCorresponding Author Nasiri SH. MD

**Keywords:** Neurometabolic disorders, children, enzymatic panels, algorithms, diagnosis

## Abstract

Neurometabolic disorders are hereditary conditions mainly affect the function of the brain and the nervous system. The prevalence of these disorders is 1 in 1,000 live births. Such disorders, at different ages, could manifest as sepsis, hypoglycemia, and other neurologic disorders. Having similar manifestations leads to delayed diagnosis of neurometabolic disorders. A number of neurometabolic disorders have known treatments; however, to prevent long-term complications the key factors are early diagnosis and treatment. Although a large number of neurometabolic diseases have no treatment or cure, the correct and on-time diagnosis before death is important for parents to have plans for prenatal diagnosis. Different diagnostic procedures could be offered to parents, enzymatic procedures, and determining metabolites in plasma, urine, and CSF, and molecular genetic diagnosis. Molecular genetic diagnostic procedures are expensive and could not be offered to all parents. Therefore, we aimed to design algorithms to diagnose neurometabolic disorders according to some frequent and characteristic signs and symptoms. By designing these algorithms and using them properly, we could offer diagnostic enzymatic panels. These enzymatic panels are inexpensive; thereby reducing the financial burden on the parents. Also, having an early diagnosis according to these panels could lead to offering more accurate and less expensive molecular genetic tests.

## Introduction

Neurometabolic diseases are a group of disorders mainly affect the brain and the nervous system. These diseases could present in all periods of life from the neonatal period to adult life. A number of neurometabolic disorders may be present after a period of normal growth and development. These disorders have different manifestations at different ages. The crude prevalence of these disorders is 1 in 1,000 live births. (1)

 Neurometabolic diseases are divided into 3 main categories. 

1. Neurometabolic diseases mainly cause toxicity of tissues.

2. Neurometabolic diseases mainly cause defective energy production.

3. Neurometabolic diseases mainly cause defective metabolism of complex molecules.

A number of neurometabolic diseases have definite treatment; however, to prevent devastating and longterm complications, the key prognostic factors are early diagnosis and treatment. To diagnose these disorders, in addition to a high index of clinical suspicion, the clinicians who deal with these disorders need to confirm the diagnosis using sophisticated genetic tests. However, genetic tests usually are delayed and expensive; therefore, many of these genetic tests could not be offered to parents. According to a number of signs and symptoms, clinicians could request a number of enzymatic tests from plasma, urine, and cerebrospinal fluid (CSF) that always are confirmatory. These enzymatic tests are also inexpensive compared to genetic tests. In this review, we aimed to design simple algorithms using several characteristic signs and symptoms. According to these algorithms, we could propose diagnostic enzymatic panels for early diagnosis in different groups of neurometabolic disorders. In every section, we begin with a characteristic sign or symptom. 

**1. Findings in neurometabolic diseases that could be approached efficiently**

**1.1 Cherry red spot**

**Figure 1:**
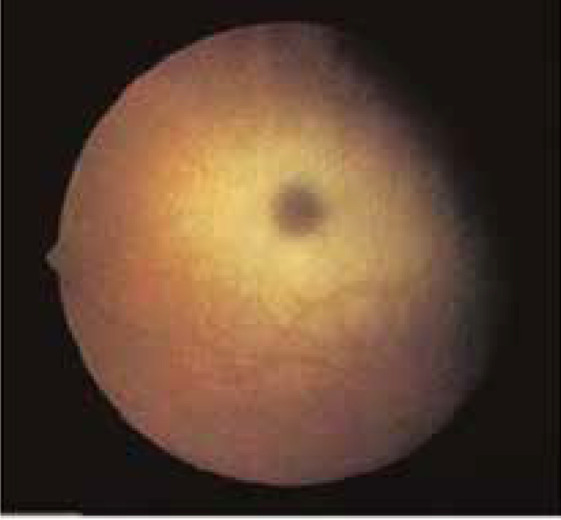
Cherry-red spot at fundoscopy

 Cherry red spot is a reddish area at the center of macula surrounded by retinal opacification. This finding could be detected in different neurometabolic disorders that affect the macular area (Figure 1). Table 1 shows these neurometabolic disorders and the involved enzymes. (2-23) According to this table and algorithm 1, enzyme measurements could be requested for early diagnosis. (3)

**Table 1:** Neurometabolic disorders with cherry-red spot and the involved enzyme

Neurometabolic disease	Defective enzyme
Tay-Sachs	Hexosaminidase A
Sandhoff	Hexosaminidase A and B
GM2 activator deficiency	Is not available
GM1 gangliosidosis	Beta-galactosidase
Niemann-pick type A	Acid sphingomyelinase
Metachromatic leukodystrophy (MLD)	Arylsulfatase A
Multiple sulfatase deficiency (MSD)	Arylsulfatase A, B, and C
Mucolipidosis type 1 (Sialidosis type II)	Alpha-neuraminidase
Mucolipidosis type II (I-cell disease)	N-acetyl glucoseamine phosphotransferase
Sialidosis type I	Alpha-neuraminidase
Galactosialidosis	Alpha-neuraminidase and BetaGalactosidase
Neuronal ceroid lipofuscinosis (NCL)	Tripeptidyl peptidase 1
Mucopolysaccharidosis (MPS) type IV and VII	N-acetylgalactosamine-6-sulfate/beta galactosidase (MPS4A/4B) and beta glucuronidase
Infantile free sialic acid storage disease (Severe form of Salla disease)	Increased free sialic acid in serum and urine and intracellular accumulation of free sialic acid in cultured fibroblasts
Farber lipogranulomatosis	Accumulation of ceramide in tissues and cultured fibroblasts

When the clinicians approach a patient who has cherry-red spot they should seek to find dysmorphism, visceromegaly, or different kinds of seizures. As shown in algorithm 1, finding dysmorphism, visceromegaly, or different kinds of seizures could help clinicians to have diagnostic plans. Brain MRI could also be used to differentiate neurometabolic disorders that show cherry-red spot. (12, 14, 16, 19, 20)

**Algorithm 1:**
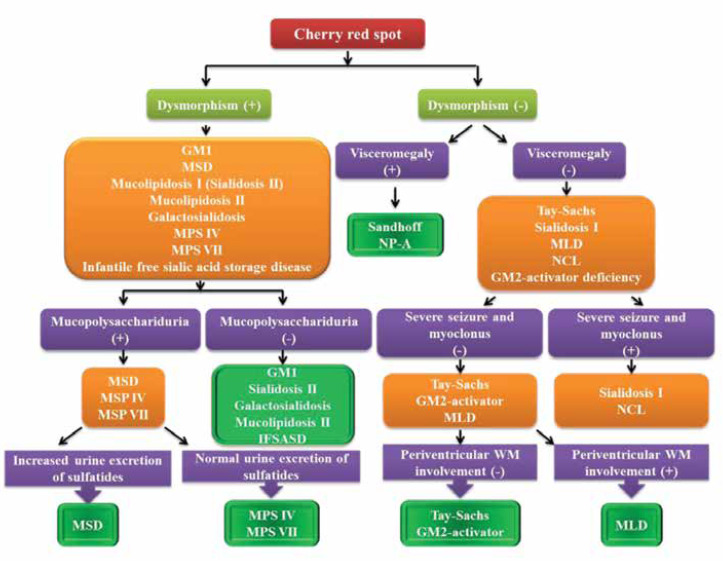
Diagnostic approach to neurometabolic disorders with a cherry-red spot

 Except for GM2 activator deficiency, the rest of neurometabolic diseases in table 1 could be diagnosed efficiently using enzyme measurement. To offer the appropriate enzymatic panel, table 1 must be reviewed precisely.

**1.2. Chronic subdural effusion and hematomas**

**Figure 2:**
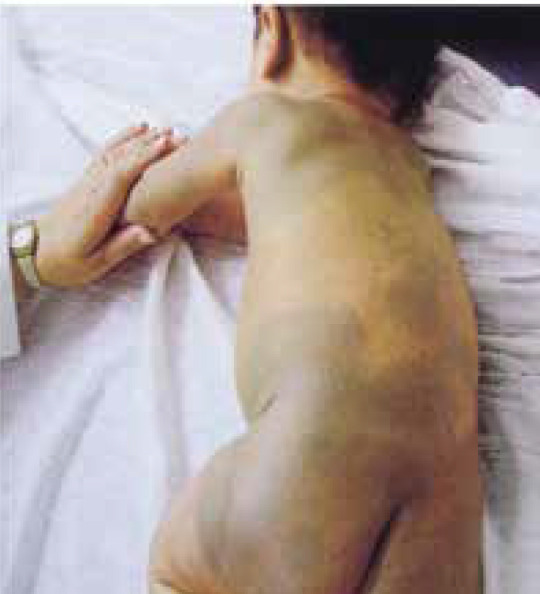
Extensive Mongolian spot

 Chronic subdural effusion and hematomas could be found in a number of neurometabolic disorders. The list of these neurometabolic disorders is not long and this finding could be approached effectively to differentiate these kinds of neurometabolic disorders (Figure 2). Table 2 shows these neurometabolic disorders and the main diagnostic procedures. (11, 24-31)

**Table 2:** Neurometabolic disorders with chronic subdural effusion and the main diagnostic approach

Neurometabolic disease	Diagnostic tests
Glutaric aciduria type 1	Urine organic acids (GC/MS^*^), Glutaryl Co dehydrogenase activity in cultured fibroblasts
Menkes disease	Plasma copper and ceruloplasmin
D2hydroxyl glutaric aciduria	Urine organic acids (GC/MS)
Pyruvate carboxylase deficiency	Plasma levels of pyruvate, lactate, and pyruvate carboxylase in cultured lymphocytes and fibroblasts
Dihydropyrimidine dehydrogenase deficiency	Not available
Biotinidase deficiency	Serum biotinidase activity

^*^GC/MS; gas chromatography/mass spectrometry

Algorithm 2 shows the diagnostic approach to neurometabolic disorders with chronic subdural effusion and hematomas. 

**Algorithm 2:**
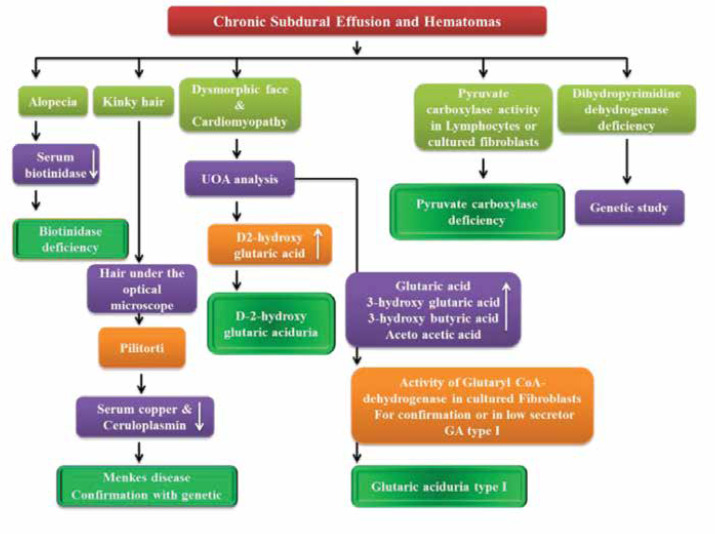
Approach to neurometabolic disorders with chronic subdural effusion and hematomas

**1.3. Alopecia and global developmental delay**

A number of neurometabolic disorders could manifest with alopecia as a definitive sign in addition to global developmental delay. (30) Many of these have curative treatment; therefore, early diagnosis is mandatory to prevent longterm complications. Table 3 shows these neurometabolic disorders and their diagnostic approach. (5, 11, 15, 30, 32)

**Table 3:** Neurometabolic diseases with alopecia and global developmental delay

Neurometabolic disease	Diagnostic approach
Hypothyroidism	T4, TSH
Vit D dependent rickets and receptor abnormalities	25-OH-Vit D and 1, 25(OH)2 vit D
Biotinidase deficiency	Biotinidase activity
Multiple carboxylase deficiency	Biotinidase, Acetyl-CoA carboxylase, Pyruvate carboxylase, MethylcrotonylCo A Carboxylase, Propionyl Co A Carboxylase

Algorithm 3 shows the diagnostic approach to neurometabolic disorders with alopecia and global developmental delay. 

**Algorithm 3.**
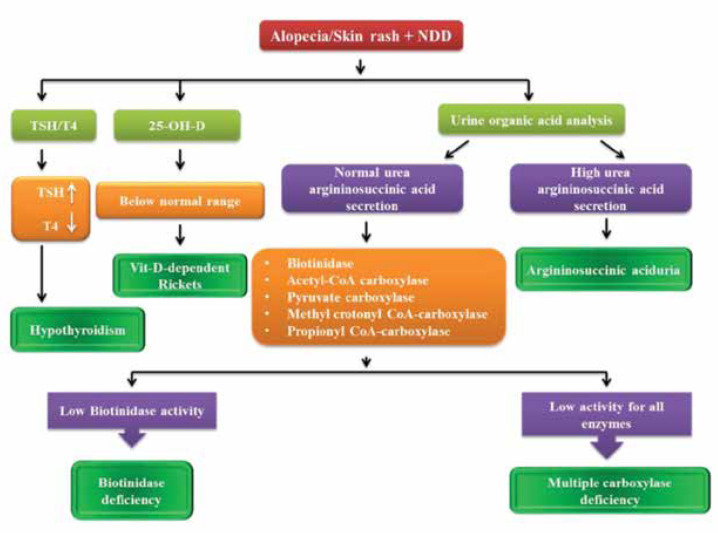
Approach to neurometabolic disorders with alopecia and global developmental delay

As shown in algorithm 3, in every patient with alopecia and global delay, we need to rule out hypothyroidism and Vit D-dependent rickets, then all we need are urine organic acid analysis using GC/MS and biotinidase activity. (29, 30)

**1.4. Extensive and long-lasting Mongolian spots (diffuse melanocytosis)**

Mongolian spots are congenital dermal melanocytoses that could normally be found on the back and the buttock regions in neonates. They disappear shortly after birth; however, when they are diffuse and extensively involve the skin, clinicians must consider a number of neurometabolic diseases (Figure 2). (17, 33) 

Table 4 shows the neurometabolic diseases with extensive Mongolian spots and the involved enzymes. 

**Table 4:** Neurometabolic disease with extensive Mongolian spots

Neurometabolic disease	Enzyme
GM1 gangliosidosis	Beta-galactosidase
Mucopolysaccharidosis (MPS) I	Alpha L iduronidase
MPS II	Iduronate 2 sulfatase
Niemann pick type A (NP A)	Alpha mannosidase
α-mannosidosis	Alpha-mannosidase
Mucolipidosis type I	Alpha-Neuraminidase
Mucolipidosis type II	N-Acetylglucosamine phosphotransferase

Algorithm 4 shows the diagnostic approach to neonates and infants with extensive Mongolian spots.

**Algorithm 4.**
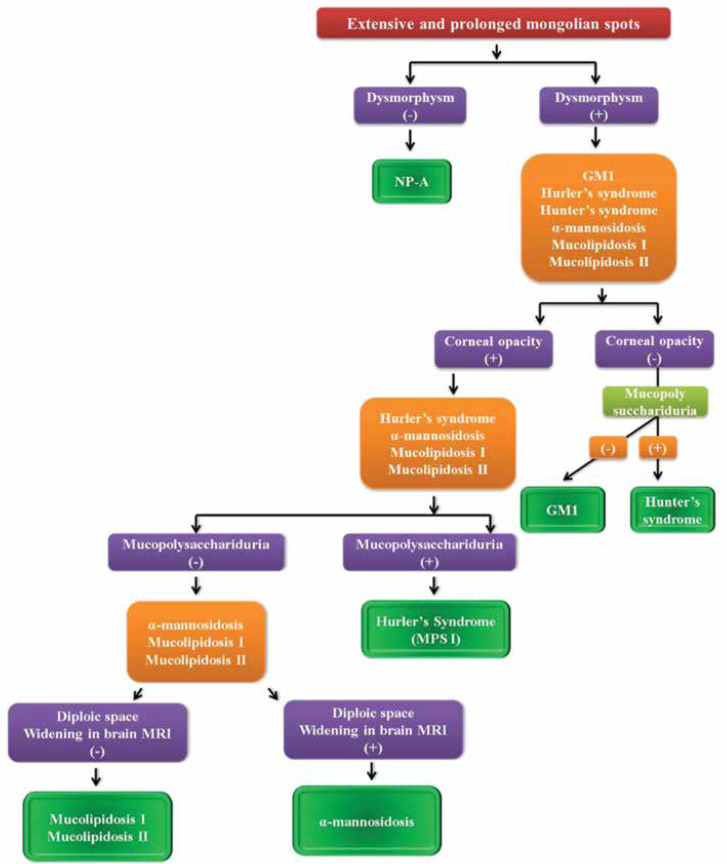
Diagnostic approach to neonates and infants with extensive Mongolian spots

As has been shown in algorithm 4, to approach extensive Mongolian spots, clinicians should seek other findings such as dysmorphism and corneal opacity. The brain MRI could also help to differentiate neurometabolic diseases with extensive Mongolian spots. 

 In the rest of this paper, we review the diagnostic approach to a number of laboratory and imaging findings that are characteristic of neurometabolic disorders.

2. Laboratory and imaging findings in neurometabolic disorders

 2.1. Hyperammonemia

Ammonia is the endproduct of amino acids’ catabolism, and its abnormal high levels are toxic to the brain and the nervous system. To excrete ammonia, mammals use the urea cycle. The urea cycle is a complex cycle that needs more than five key enzymes to work efficiently and to transform produced ammonia to less toxic metabolites in the body. Measurement of ammonia level is mandatory in every patient with encephalopathy who might suffer from neurometabolic disorders. Neonates with hyperammonemia could present with progressive lethargy, vomiting, hypotonia, and seizures mainly after feeding. In older infants and children, hyperammonemia could present with ataxia, decreased consciousness, agitation, and irritability finally, might lead to coma. In all patients with unexplained encephalopathy, serum ammonia should be measured as soon as possible. Table 5 shows the main causes of hyperammonemia and the involved enzymes. (1, 11, 27, 34)

**Table 5.** Neurometabolic disorders with hyperammonemia and the involved enzymes

Neurometabolic disorder	Enzyme
Urea cycle defects	Carbamyl phosphate synthetase (CPS) deficiency, Ornithine transcarbamylase (OTC) deficiency, Argininosuccinate synthetase (AS) deficiency, Argininosuccinate lyase (AL) deficiency, Arginase deficiency?, Nacetyl glutamate synthetase deficiency
Organic acidemias	Propionic academia, Methylmalonic academia, Isovaleric academia, Beta-ketothiolase deficiency, Multiple carboxylase deficiency, Glutaric aciduria type II, 3-hydroxy -3-methylglutaric aciduria
Fatty acid oxidation disorders	Long Chain Acyl CoA Dehydrogenase Deficiency (LCAD) Medium Chain Acyl CoA Dehydrogenase Deficiency (MCAD)

Other etiologies of hyperammonemia are pyruvate dehydrogenase deficiency, lysinuric protein intolerance (LPI), Hyperammonemia-Hyperornithinemi Homocitrullinemia syndrome (HHH), transient hyperammonemia of neonate, and congenital hyperinsulinism hyperammonemia. Algorithm 5 shows the diagnostic approach in patients with hyperammonemia. 

**Algorithm 5:**
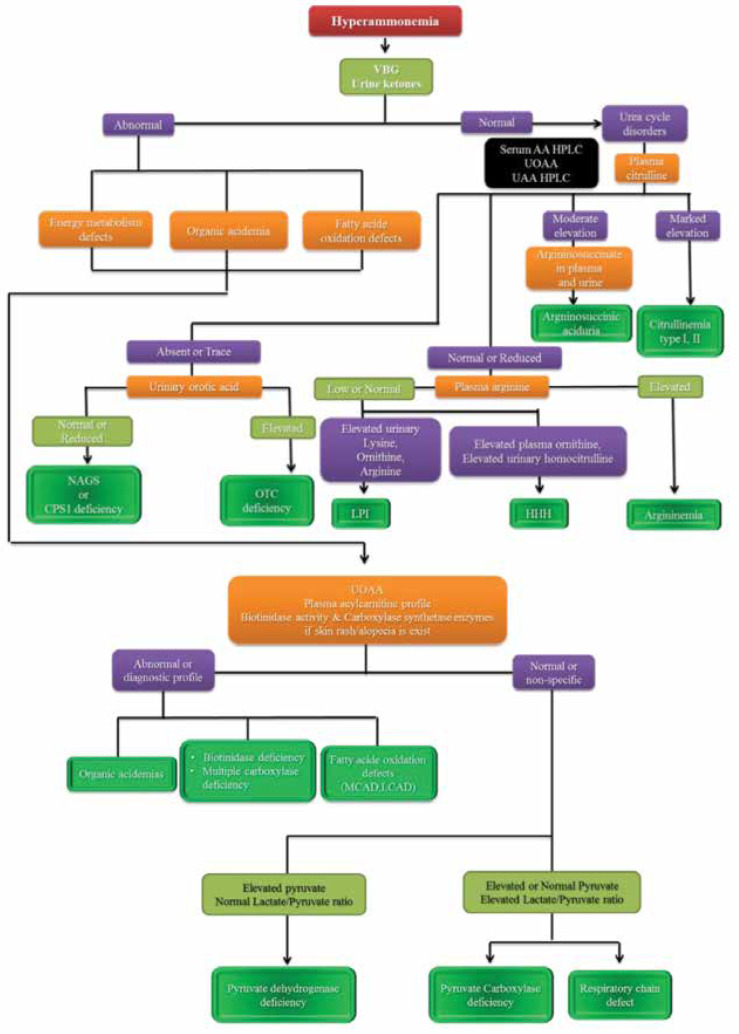
Diagnostic approach in patients with hyperammonemia

**Hypertyrosinemia**

Tyrosine is the precursor for a bunch of neurotransmitters such as dopamine, norepinephrine, and epinephrine. It is also found in the structure of thyroxin and melanin. A number of congenital and acquired disorders cause hypertyrosinemia such as medications (amiodarone), liver dysfunction, hypothyroidism, high protein diet, and neurometabolic disorders. Algorithm 6 shows the diagnostic approach to a patient with hypertyrosinemia. (32)

**Algorithm 6:**
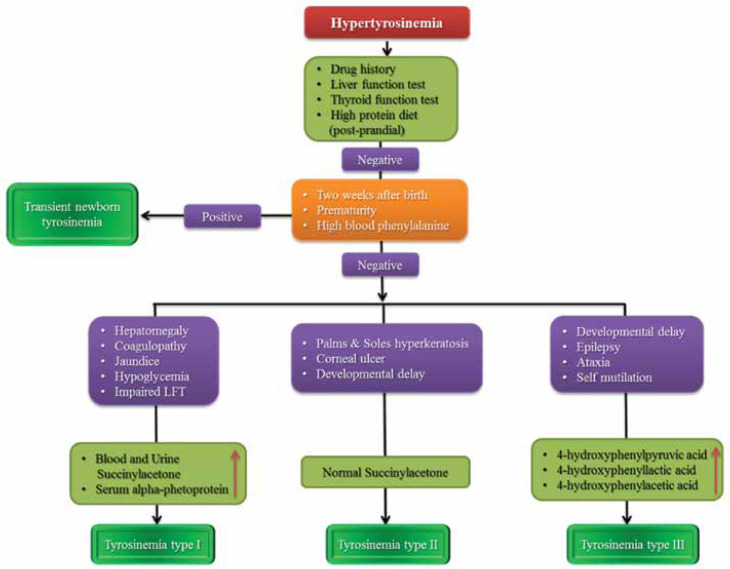
Diagnostic approach to a patient with hypertyrosinemia

**In conclusion, **In this short review, we showed that many neurometabolic disorders could be simply diagnosed by having a diagnostic plan after finding cherry-red spot, alopecia and global delay, and extensive Mongolian spot in the neurologic examination of the patient. We also showed diagnostic algorithms in patients with hyperammonemia, hypertyrosinemia, bilateral striatal necrosis. By using these algorithms, almost all clinicians could precisely approach to different kinds of neurometabolic disorders and request inexpensive enzymatic panels to administer early treatment. Using these algorithms also help clinicians dealing with prenatal consults with parents. 

## References

[1] Filiano JJ (2006). Neurometabolic diseases in the newborn. Clin Perinatol..

[2] Fernandes Filho JA, Shapiro BE (2004). Tay-Sachs disease. Arch Neurol..

[3] Suvarna JC, Hajela SA (2008). Cherry-red spot. J Postgrad Med..

[4] Gieselmann V, Franken S, Klein D, Mansson JE, Sandhoff R, Lullmann Rauch R (2003). Metachromatic leukodystrophy: consequences of sulphatide accumulation. Acta Paediatr Suppl..

[5] Dierks T, Schlotawa L, Frese MA, Radhakrishnan K, von Figura K, Schmidt B (2009). Molecular basis of multiple sulfatase deficiency, mucolipidosis II/III and Niemann-Pick C1 disease - Lysosomal storage disorders caused by defects of non-lysosomal proteins. Biochim Biophys Acta..

[6] Jenkins RW, Canals D, Hannun YA (2009). Roles and regulation of secretory and lysosomal acid sphingomyelinase. Cell Signal..

[7] Lynch DT, Czuchlewski DR (2016). Peripheral blood findings in GM1 gangliosidosis. Blood..

[8] Pshezhetsky AV, Ashmarina M (2001). Lysosomal multienzyme complex: biochemistry, genetics, and molecular pathophysiology. Prog Nucleic Acid Res Mol Biol..

[9] Santos-Lozano A, Villamandos Garcia D, Sanchis-Gomar F, Fiuza-Luces C, Pareja-Galeano H, Garatachea N (2015). Niemann-Pick disease treatment: a systematic review of clinical trials. Ann Transl Med..

[10] Gieselmann V (2003). Metachromatic leukodystrophy: recent research developments. J Child Neurol..

[11] Fuchshuber A, Suormala T, Roth B, Duran M, Michalk D, Baumgartner ER (1993). Holocarboxylase synthetase deficiency: early diagnosis and management of a new case. Eur J Pediatr..

[12] Altarescu G, Sun M, Moore DF, Smith JA, Wiggs EA, Solomon BI (2002). The neurogenetics of mucolipidosis type IV. Neurology..

[13] Caciotti A, Di Rocco M, Filocamo M, Grossi S, Traverso F, d'Azzo A (2009). Type II sialidosis: review of the clinical spectrum and identification of a new splicing defect with chitotriosidase assessment in two patients. J Neurol..

[14] Itoh K, Naganawa Y, Matsuzawa F, Aikawa S, Doi H, Sasagasako N (2002). Novel missense mutations in the human lysosomal sialidase gene in sialidosis patients and prediction of structural alterations of mutant enzymes. J Hum Genet..

[15] Lin MH, Pitukcheewanont P (2012). Mucolipidosis type II (I-cell disease) masquerading as rickets: two case reports and review of literature. J Pediatr Endocrinol Metab..

[16] Matheus MG, Castillo M, Smith JK, Armao D, Towle D, Muenzer J (2004). Brain MRI findings in patients with mucopolysaccharidosis types I and II and mild clinical presentation. Neuroradiology..

[17] Schwartz IV, Ribeiro MG, Mota JG, Toralles MB, Correia P, Horovitz D (2007). A clinical study of 77 patients with mucopolysaccharidosis type II. Acta Paediatr..

[18] Wraith JE, Scarpa M, Beck M, Bodamer OA, De Meirleir L, Guffon N (2008). Mucopolysaccharidosis type II (Hunter syndrome): a clinical review and recommendations for treatment in the era of enzyme replacement therapy. Eur J Pediatr..

[19] Achyuthan KE, Achyuthan AM (2001). Comparative enzymology, biochemistry and pathophysiology of human exo-alpha-sialidases (neuraminidases). Comp Biochem Physiol B Biochem Mol Biol..

[20] Ruivo R, Anne C, Sagne C, Gasnier B (2009). Molecular and cellular basis of lysosomal transmembrane protein dysfunction. Biochim Biophys Acta..

[21] Park JH, Schuchman EH (2006). Acid ceramidase and human disease. Biochim Biophys Acta..

[22] Cooper JD (2003). Progress towards understanding the neurobiology of Batten disease or neuronal ceroid lipofuscinosis. Curr Opin Neurol..

[23] Mondal RK, Nandi M, Datta S, Hira M (2009). Disseminated lipogranulomatosis. Indian Pediatr..

[24] Strauss KA, Puffenberger EG, Robinson DL, Morton DH (2003). Type I glutaric aciduria, part 1: natural history of 77 patients. Am J Med Genet C Semin Med Genet..

[25] Naganawa Y, Itoh K, Shimmoto M, Takiguchi K, Doi H, Nishizawa Y (2000). Molecular and structural studies of Japanese patients with sialidosis type 1. J Hum Genet..

[26] Kaler SG, Holmes CS, Goldstein DS, Tang J, Godwin SC, Donsante A (2008). Neonatal diagnosis and treatment of Menkes disease. N Engl J Med..

[27] Santarelli F, Cassanello M, Enea A, Poma F, D'Onofrio V, Guala G (2013). A neonatal case of 3-hydroxy-3-methylglutaric-coenzyme A lyase deficiency. Ital J Pediatr..

[28] Wang D, Yang H, De Braganca KC, Lu J, Yu Shih L, Briones P (2008). The molecular basis of pyruvate carboxylase deficiency: mosaicism correlates with prolonged survival. Mol Genet Metab..

[29] Wolf B (2016). Biotinidase deficiency and our champagne legacy. Gene..

[30] Wolf B, Grier RE, Allen RJ, Goodman SI, Kien CL (1983). Biotinidase deficiency: the enzymatic defect in late-onset multiple carboxylase deficiency. Clin Chim Acta..

[31] Lindner M, Kolker S, Schulze A, Christensen E, Greenberg CR, Hoffmann GF (2004). Neonatal screening for glutaryl-CoA dehydrogenase deficiency. J Inherit Metab Dis..

[32] Russo PA, Mitchell GA, Tanguay RM (2001). Tyrosinemia: a review. Pediatr Dev Pathol..

[33] Borgwardt L, Lund AM, Dali CI (2014). Alpha-mannosidosis - a review of genetic, clinical findings and options of treatment. Pediatr Endocrinol Rev..

[34] Kleijer WJ, Garritsen VH, Linnebank M, Mooyer P, Huijmans JG, Mustonen A (2002). Clinical, enzymatic, and molecular genetic characterization of a biochemical variant type of argininosuccinic aciduria: prenatal and postnatal diagnosis in five unrelated families. J Inherit Metab Dis..

